# The Implications of Incongruence between Gene Tree and Species Tree Topologies for Divergence Time Estimation

**DOI:** 10.1093/sysbio/syac012

**Published:** 2022-02-15

**Authors:** Tom Carruthers, Miao Sun, William J Baker, Stephen A Smith, Jurriaan M de Vos, Wolf L Eiserhardt

**Affiliations:** Royal Botanic Gardens, Kew, Richmond, Surrey TW9 3AE, UK; Department of Biology, Aarhus University, 8000 Aarhus C, Denmark; Royal Botanic Gardens, Kew, Richmond, Surrey TW9 3AE, UK; Department of Ecology and Evolutionary Biology, University of Michigan, Ann Arbor, Michigan, 48109, USA; Department of Environmental Sciences – Botany, University of Basel, 4056 Basel, Switzerland; Royal Botanic Gardens, Kew, Richmond, Surrey TW9 3AE, UK; Department of Biology, Aarhus University, 8000 Aarhus C, Denmark

## Abstract

Phylogenetic analyses are increasingly being performed with data sets that incorporate hundreds of loci. Due to incomplete lineage sorting, hybridization, and horizontal gene transfer, the gene trees for these loci may often have topologies that differ from each other and from the species tree. The effect of these topological incongruences on divergence time estimation has not been fully investigated. Using a series of simulation experiments and empirical analyses, we demonstrate that when topological incongruence between gene trees and the species tree is not accounted for, the temporal duration of branches in regions of the species tree that are affected by incongruence is underestimated, whilst the duration of other branches is considerably overestimated. This effect becomes more pronounced with higher levels of topological incongruence. We show that this pattern results from the erroneous estimation of the number of substitutions along branches in the species tree, although the effect is modulated by the assumptions inherent to divergence time estimation, such as those relating to the fossil record or among-branch-substitution-rate variation. By only analyzing loci with gene trees that are topologically congruent with the species tree, or only taking into account the branches from each gene tree that are topologically congruent with the species tree, we demonstrate that the effects of topological incongruence can be ameliorated. Nonetheless, even when topologically congruent gene trees or topologically congruent branches are selected, error in divergence time estimates remains. This stems from temporal incongruences between divergence times in species trees and divergence times in gene trees, and more importantly, the difficulty of incorporating necessary assumptions for divergence time estimation. [Divergence time estimation; gene trees; species tree; topological incongruence.]

Divergence time estimation provides a basis for determining the ages of different clades, the rates that different clades have diversified, absolute rates of nucleotide substitution, and the intrinsic and extrinsic factors that have affected these patterns (Baldwin and Sanderson 1998; Hughes and Eastwood 2006; Simon et al. 2009; Särkinen et al. 2012; Magallón et al. 2015; Lagomarsino et al. 2016; Folk et al. 2019; Muñoz-Rodríguez et al. 2018, 2019; Sun et al. 2020). Interest in divergence time estimation arose from the proposal of the molecular clock hypothesis by Zuckerkandl and Pauling (1962, 1965), which describes how the degree of divergence between any pair of molecular sequences is correlated with the time since they diverged. Since then, methods have developed that attempt to account for the complexity of ways that molecular sequences evolve—with a particular focus being on how best to account for differences in substitution rates ($r$) among lineages (Sanderson 1997, 2002; Thorne et al. 1998; Kishino et al. 2001; Drummond et al. 2006; dos Reis et al. 2014; Lartillot et al. 2016).

It is now common to use data sets of hundreds of loci in phylogenetic inference and divergence time estimation, and because of this, new methodological challenges have arisen. One of the most important of these is the expectation that phylogenies for individual loci (gene trees) may differ topologically from each other, and also differ topologically from the species tree (Degnan and Rosenberg 2009). Topological incongruence between gene trees and the species tree has received extensive attention in relation to phylogenetic inference. For example, it has been shown that a failure to account for topological incongruence by assuming that all gene trees conform to the same topology and analyzing them as a single concatenated alignment, can jeopardize estimates of phylogenetic relationships (Degnan and Rosenberg 2009; Hahn and Nakhleh 2016; Mirarab et al. 2014; Mendes and Hahn 2016; Copetti et al. 2017; Walker et al. 2021).

##  

### Topological Incongruence between Gene Trees and the Species Tree May Have Important Effects on Divergence Time Estimates

The effect of topological incongruence between gene trees and the species tree has received considerably less attention in relation to divergence time estimation, although several studies have highlighted patterns that are directly relevant. For example, despite not focusing explicitly on divergence time estimation, Mendes and Hahn (2016) demonstrated that estimation of the number of molecular substitutions ($n$) along branches in a species tree is biased by topological incongruence. First, they noted that topological incongruence between gene trees and the species tree caused an increase in the total estimated $n$ across the species tree. This is because substitutions that have evolved on topologically incongruent gene trees must be estimated to have occurred several times when estimation of $n$ is performed with the species tree topology. Second, they showed that for branches in the species tree that were not represented in a gene tree, estimates of $n$ were lower (Mendes and Hahn 2016). This is because substitutions cannot be estimated for branches in the species tree that do not exist in an underlying gene tree.

The findings of Mendes and Hahn (2016) are relevant to divergence time estimation because the temporal duration of a branch ($t$) is equal to the number of substitutions for the branch divided by the substitution rate for the branch, $\frac{n}{r}$. As such, it has previously been highlighted that the patterns discovered by Mendes and Hahn (2016) may have had important effects on divergence time estimates in birds (Suh 2016) and estimates of $r$ in great apes (Mello and Schrago 2019). Further, even though Mendes and Hahn (2016) primarily focused on the analysis of individual loci, it is likely their findings are relevant to how sequence data are analyzed in the majority of divergence time analyses that are based on a concatenated alignment of multiple loci and a single species tree topology. Specifically, if a percentage of gene trees for loci in the concatenated alignment are topologically incongruent with the species tree, substitutions for these loci are likely to be estimated several times on the species tree, leading to inflation of the total estimated $n$ across the species tree (Fig. 1). Meanwhile, for branches in the species tree that are not represented by the topologically incongruent gene trees, substitutions cannot occur at the relevant loci meaning that $n$ for these branches is likely to be underestimated (Fig. 1).

**Figure 1.**
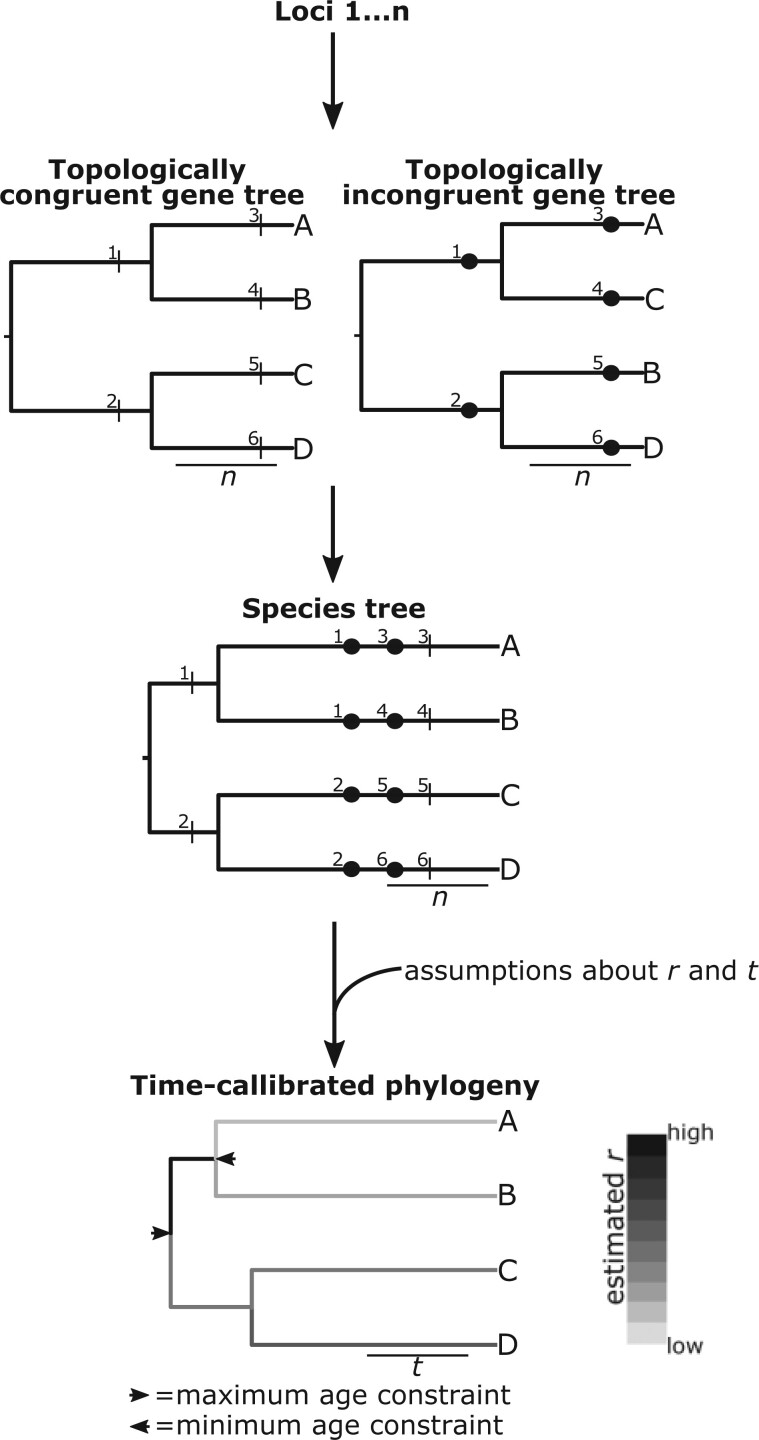
An extension of Figure 1 from Mendes and Hahn (2016), illustrating the implications of topological incongruence between gene trees and the species tree for branch-specific parameter estimation in the species tree. Loci $1\ldots n$ are sampled for four taxa, A, B, C, and D. Some gene trees for these loci are topologically congruent with the species tree, whilst some gene trees are topologically incongruent. These two alternatives are shown. Substitutions occur along the branches of the gene trees. These are indicated by numbered black dashes in the topologically congruent gene trees and numbered black circles in the topologically incongruent gene trees. If the loci are concatenated and used to estimate branch lengths (in units of $n$) in the species tree, substitutions in the incongruent gene trees on branches leading to the clade of AC or BD are likely to be estimated to have occurred twice in the species tree and placed on the terminals. This may cause overestimation of $n$ for terminals in the species tree. Alternatively, substitutions in the topologically incongruent gene trees are unlikely to be estimated to have occurred on the branches leading to AB and CD in the species tree. As such, estimates of $n$ for these branches in the species tree are likely to be reduced. This pattern is consistent with the findings of Mendes and Hahn (2016). These effects on estimates of $n$ are likely to affect estimates of $t$ and $r$ when estimating divergence times. However, the nature of assumptions that are required when estimating divergence times are likely to modulate this effect.

Nevertheless, the nature of the relationship between the findings of Mendes and Hahn (2016) and consequent effects on $t$ and $r$ remains unclear. Importantly, when analyzing molecular sequence data, only $n$ is inferred directly from the data, which in turn represents the product of $r$ and $t$. Assumptions are therefore required for estimating $r$ and $t$, such as the nature of variation in $r$ among different lineages (Sanderson 1997, 2002; Drummond et al. 2006; Drummond and Suchard 2011; Lartillot et al. 2016) and the manner by which fossil occurrences correspond to the ages of different clades (Donoghue and Benton 2007; Ho and Phillips 2009; Heath et al. 2014). Importantly, these assumptions underpin the estimated parameter values in a divergence time analysis (Magallón et al. 2013; Brown and Smith 2018; Carruthers and Scotland 2020, 2021) (Fig. 1). Further complexity also stems from the fact that topological incongruence affects parameter estimation in a branch-specific manner (Fig. 1). The combination of nonidentifiable parameters with branch-specific patterns has already been shown to make divergence time estimation one of the most challenging endeavors in systematic biology (Sanderson 1997; Britton 2005; Magallón et al. 2013; Carruthers and Scotland 2020). Topological incongruence therefore has the potential to accentuate this problem.

Aside from Mendes and Hahn (2016), several other studies have highlighted important patterns concerning the implications of differences between gene trees and the species tree for divergence time estimation. Primarily, these studies have focused on the fact that in a multispecies coalescent process, divergences in gene trees necessarily predate those in the species tree, such that when this issue is not accounted for, species divergence times are overestimated (McCormack et al. 2010; Angelis and dos Reis 2015; Stange et al. 2018). Although not strictly related to topological incongruence, these studies demonstrate how in a biological context in which topological incongruence can be generated (a multispecies coalescent process), other phenomena will also influence divergence time estimates. This highlights a more general and important point that the implications of topological incongruence for divergence time estimation are likely to be modulated by the context in which incongruence occurs. This includes the biological context, such as the processes that generated topological incongruence and the characteristics of the species tree (size or whether it is balanced or imbalanced). It also includes the methodological context, such as the extent to which error or uncertainty in phylogenetic inference affects the perception of topological incongruence between gene trees and the species tree.

In addition to these theoretical studies, recent contributions (Jarvis et al. 2014; Doyle et al. 2015; Smith et al. 2018) have also set out approaches for a critical evaluation of the loci that are included in divergence time analyses such that the effects of topological incongruence between gene trees and the species tree are ameliorated. These methods are centered on selecting loci based on their conformity to a strict molecular clock and the degree of topological congruence between gene trees and the species tree, and they have been shown to reduce error in divergence time estimates in the species tree.

### Characterizing the Effects of Topological Incongruence on Divergence Time Estimation

Here, we undertake a comprehensive study of the implications of topological incongruence between gene trees and the species tree for divergence time estimation. We address this problem from a theoretical perspective and with respect to the effectiveness of methods designed to lighten the burden of topological incongruence on divergence time estimates. To fulfill these aims, we use simulation experiments to outline key principles, and analyses of a seed plant data set (comprising 101 taxa sampled for 351 single-or low-copy nuclear loci) to determine the relevance of these principles in an empirical system.

In this study, topological incongruence for a branch in the species tree occurs when a gene tree does not possess a branch with the same descendant clade as the branch in the species tree. Each branch in the species tree therefore has a value for topological incongruence that corresponds to the percentage of gene trees that do not possess a branch with the same descendant clade, and terminal branches cannot be affected by incongruence. There is additional complexity in larger species trees because a gene tree can be incongruent for a branch in the species tree to different degrees, depending on the proportion of terminals that differ between the gene tree and species tree for a given clade in the species tree (Fig. 2). Importantly, this study also refers explicitly to topological incongruence resulting from biological processes (such as incomplete lineage sorting, hybridization, or horizontal gene transfer), rather than apparent topological incongruence resulting from error in gene tree or species tree estimation.

**Figure 2.**
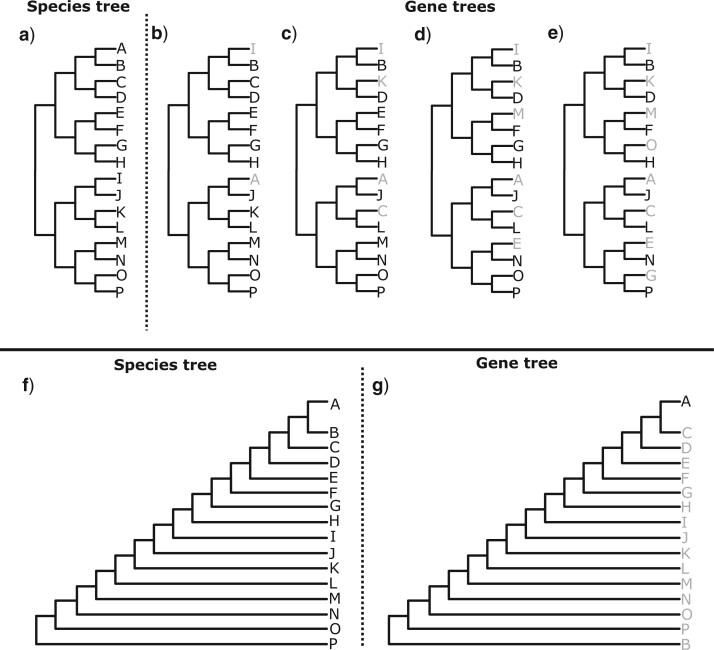
An illustration of how topological incongruence for a branch in the species tree is affected by the proportion of tips that differ in a gene tree for a given clade in the species tree. a) A balanced species tree is shown, b–e) gene trees that have increasing levels of topological incongruence with the species tree are shown. Tips that differ in their phylogenetic placement in the gene tree relative to the species tree are shown in gray. Note how for a given clade in the species tree, the proportion of tips that differ between the gene tree and species tree increases from b) to e). f) An imbalanced species tree is shown, and g) a topologically incongruent gene tree is shown. Tips are colored as with the gene trees in b–e. Note how for a given clade in the species tree the proportion of tips that differ between the species tree and gene tree varies.

#### A general theoretical overview of the implications of topological incongruence

The study will first address the following interrelated theoretical questions: 1) How does the mathematical relationship between $n$, $r$, and $t$ underpin the effect of topological incongruence on divergence time estimates? 2) How is this effect modulated by the necessary methodological assumptions in divergence time estimation? 3) How is this effect modulated by the level of topological incongruence in the data set? 4) How is this effect modulated by the way in which topological incongruence is generated? 5) How is this effect modulated by species trees of different sizes and characteristics (balanced or imbalanced)?

#### Evaluating methods for alleviating the effects of topological incongruence

The effectiveness of three different methods for alleviating the effect of topological incongruence will then be evaluated. First, only subsets of loci with gene trees that are topologically congruent with the species tree will be incorporated into divergence time estimation (Fig. 3a and b). This approach is analogous to “gene shopping” as set out by Smith et al. (2018) but focuses specifically on selecting loci with topologically congruent gene trees. Second, parameter estimates are derived for branches in the species tree (Fig. 3a) by determining the mean parameter value for the branch across all gene trees that possess an equivalent branch (Fig. 3c). With this method, information from gene trees that are topologically incongruent with the species tree can be used if they have a subset of branches that are topologically congruent with the species tree. Third, divergence times will be estimated in a multispecies coalescent framework such that topological incongruence is explicitly accounted for.

**Figure 3.**
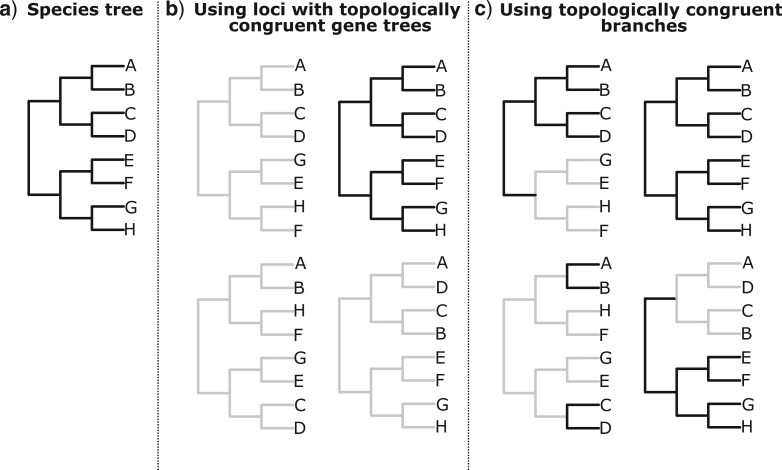
A summary of different potential methods for accounting for topological incongruence between gene trees and the species tree. For a given species tree (a), parameter estimates could be made by selecting loci with gene trees that are topologically congruent with the species tree (b) or selecting individual branches from gene trees that are topologically congruent with the species tree (c). For b) and c), topologically congruent gene trees or branches are shown in black.

## Materials and Methods

### Generating Simulated Data Sets

#### Simple four-taxon simulations

These simulations were designed to enable topological incongruence to be evaluated in the simplest possible context, where individual gene trees are either congruent or incongruent with the species tree topology (there are no degrees of incongruence depending on the proportion of tips that differ for a given clade), and no other processes occur that are likely to bias divergence time estimates (such as gene tree divergence times predating species tree divergence times). The four-taxon species tree consisted of taxa A, B, C, and D. In this species tree, A and B formed a clade and C and D formed a clade. The root age was 1.0, and the ages of the two other divergence times (between A and B, and C and D) were 0.5.

For this species tree, six sets of 400 gene trees were simulated. For each set of gene trees, the topology of a percentage of the gene trees (either 0, 10, 20, 30, 40, or 50) was incongruent with that of the species tree such that A and C formed a clade and B and D formed a clade (Fig. 1). For the incongruent gene trees, the root age was still 1.0, and the two other divergence times were still 0.5. The simSeq function in phangorn (Schliep 2011) was used to simulate sequences of 800 base pairs (bp) along the branches of each gene tree according to a Jukes–Cantor (JC) model with $r = 0.05$.

#### Simple 16-taxon simulations

These simulations enabled the implications of topological incongruence to be determined in larger phylogenies where the species tree was either perfectly balanced (Fig. 2a) or perfectly imbalanced (Fig. 2f). In each case, the species tree topology conformed to Figure 2a or f. For the balanced tree, $t$ for all branches in the species tree was 0.2. For the imbalanced tree, $t$ for all internal branches in the species tree was 0.2, but for terminal branches it became sequentially longer, increasing from 0.2 to 3.2.

For the balanced tree, five sets of 400 gene trees were simulated. In the first set, all gene trees were topologically congruent with the species tree. In the second set, 50$\%$ of gene trees were topologically incongruent, although the incongruent gene trees only differed from the species tree in the phylogenetic placement of two terminals, as displayed in Figure 2b. In the third set, 50$\%$ of gene trees were topologically incongruent and differed from the species tree in the phylogenetic placement of four terminals, as displayed in Figure 2c. In the fourth set, 50$\%$ of gene trees were topologically incongruent and differed from the species tree in the phylogenetic placement of six terminals, as displayed in Figure 2d. In the fifth set, 50$\%$ of gene trees were topologically incongruent and differed from the species tree in the phylogenetic placement of eight terminals, as displayed in Figure 2e. For the imbalanced tree, one set of 400 gene trees was simulated in which 50$\%$ of gene trees were incongruent with the species tree, as displayed in Figure 2g. In each case, molecular sequences were simulated along the branches of the gene trees, as outlined in the simple four-taxon example above.

#### Multispecies coalescent simulation

A further set of simulations was designed to enable the implications of topological incongruence to be evaluated in the context of a multispecies coalescent process. The relevance of results derived from the simple simulations could therefore be examined in a more complex setting where other processes are also likely to affect parameter estimates, such as divergence times in gene trees predating those in the species tree. The same two species trees as in the simple 16-taxon example above were used (Fig. 2a and f). Four hundred gene trees were then simulated for each species tree according to a multispecies coalescent process with an effective population size (*Ne*) equal to 0.12. For each (internal) branch in the species tree, this resulted in an average of 87$\%$ of gene trees being topologically congruent. For each gene tree, sequences were simulated as outlined above.

In a further simulation, multispecies coalescent data sets were generated according to a four-taxon species tree. This was to enable greater computational tractability when $t$ was subsequently estimated explicitly within a multispecies coalescent framework (see below). In this case, the topology of the species tree was the same as in the simple four-taxon simulation, but for each branch $t = 0.2$ (as in the multispecies coalescent 16-taxon simulation). Gene trees and molecular sequences were simulated according to this species tree and with the same parameters as the multispecies coalescent 16-taxon simulation.

### A General Theoretical Overview of the Implications of Topological Incongruence

The simulated data sets were used to estimate $n$, $t$, and $r$ for branches in the species tree. The implications of topological incongruence for parameter estimation could therefore be determined (Table 1). Parameters were estimated with concatenated alignments from each simulation, and with the species tree being fixed to the “correct” topology (i.e., the topology that was used when simulating the data). All analyses were performed in RevBayes (Höhna et al. 2016) and parameter estimates were compared to the “correct” simulated values.

**Table 1.** A summary of the different analyses performed in this study.

Purpose	Data	Inference method
A general theoretical overview of the implications of topological incongruence	Simple four-taxon simulation	All loci concatenated
	Simple 16-taxon simulation	All loci concatenated
	Multispecies coalescent 16-taxon simulation	All loci concatenated
Evaluating methods for alleviating the effects of topological incongruence	Simple four-taxon simulation	Loci with topologically congruent gene trees concatenated
		Congruent branches
		Multispecies coalescent
	Multispecies coalescent 16-taxon simulation	Loci with topologically congruent gene trees concatenated
		Congruent branches
	Multispecies coalescent four-taxon simulation	Multispecies coalescent
Determine the implications of topological incongruence in an empirical data set	Angiosperms 353 loci	All loci concatenated$^{a}$
		Loci with topologically congruent gene trees concatenated$^{\rm a}$
		Congruent branches$^{\rm a}$

$^{\rm a}$ In each case, analyses were performed with a smoothing value of 10,000 and no internal fossil calibrations *or* a smoothing value selected by cross-validation and internal fossil calibrations.

For estimating $t$, a Yule tree prior was used with the root node age being fixed to that of the species tree from the simulation (1 for the simple four-taxon simulation, balanced simple 16-taxon simulation, and balanced multispecies coalescent 16-taxon simulation; or 3.2 for the imbalanced simple 16-taxon simulation, and imbalanced multispecies coalescent simulation). There were no further temporal constraints at internal nodes. A JC model of sequence evolution was used with a strict clock. For data from the simple four-taxon and simple 16-taxon simulations, $r$ for the strict clock was 0.05, the value under which $r$ was simulated. For data from the multispecies coalescent simulation, $r$ for the strict clock was sampled from a uniform distribution spanning 1$e^{-6}$ to 1. This is because the simulated gene trees have evolved over a longer time than the simulated species tree that is used to inform the root node age constraint when estimating $t$. As such, constraining $r$ for the strict clock to 0.05 would likely lead to additional biases that are independent of topological incongruence and not the focus of this study.

When estimating $n$ and $r$, only the data sets from the simple four-taxon simulations and simple 16-taxon simulations were used. This is because the more complex multispecies coalescent simulation incorporates additional phenomena that are not directly relevant to topological incongruence (such as divergences in gene trees predating those in the species tree), and the purpose of the analysis based on the multispecies coalescent simulation was simply to determine whether key patterns concerning the estimation of $t$ from the simple simulations were relevant in this more complex setting.

When estimating $n$, an unrooted tree prior was used alongside a JC model of sequence evolution. When estimating $r$, two sets of analyses were performed. In the first case, an uncorrelated lognormal (UCLN) relaxed clock with $m = 5e^{-2}$ and $v = 0.001$ was used (subsequently referred to as relaxed clock with low variance), and in the second case, a UCLN relaxed clock with $m = 5e^{-2}$ and $v = 0.1$ was used (subsequently referred to as relaxed clock with high variance). In both cases, a Yule tree prior was used with a JC model of sequence evolution, and all divergence times were fixed to the correct value. The purpose of the analyses for estimating $r$ was to specifically characterize the behavior of a relaxed clock model (a widely used tool for linking $n$, $t$, and $r$) in the context of topological incongruence and determine how the behavior of the model was influenced by its assumptions without additional effects resulting from erroneous estimates of $t$. For estimates of $n$, $t$, and $r$, parameter estimates were compared to the “correct” simulated values.

### Evaluating Methods for Alleviating the Effects of Topological Incongruence

These analyses were performed with data from the simple four-taxon simulation in which 50$\%$ of gene trees were topologically incongruent with the species tree, and with data from the multispecies coalescent simulations (either the balanced multispecies coalescent 16-taxon simulation, or, when analyzing data in a multispecies coalescent framework the multispecies coalescent four-taxon simulation). The potential effectiveness of each method could therefore be clarified in the most-simple case, before evaluating it in the most complex case that incorporates more biologically realistic processes (Table 1) In this section, $t$ was estimated from only those loci with gene trees that were topologically congruent with the species tree (Fig. 3a and b), only the branches in gene trees that were topologically congruent with the species tree (Fig. 3a and c), and in a multispecies coalescent framework.

#### Estimating $t$ with loci derived from gene trees that are topologically congruent with the species tree

For data from the simple four-taxon simulation, loci were selected for which the gene trees were entirely topologically congruent with the species tree. These loci were concatenated and used to estimate $t$ in the species tree. Aside from filtering for loci with topologically congruent gene trees, the same methods were implemented as when the entire data set was analyzed.

For data from the multispecies coalescent 16-taxon simulation, initial analyses also involved selecting only loci with gene trees that were entirely topologically congruent with the species tree, concatenating the loci, and estimating $t$ in the species tree. However, in the multispecies coalescent 16-taxon simulations, topologically incongruent gene trees varied in the extent to which they were topologically incongruent with the species tree. Therefore, $t$ was also estimated from loci with topologically congruent gene trees, plus subsets of increasingly incongruent gene trees (with either 1, 2, 3, or 4 branches that were topologically incongruent with the species tree). This provided further insight into the effect of different levels of topological incongruence on estimates of $t$ in the species tree, as well as the trade-off between analyzing less data and reducing the extent of topological incongruence within the data set. As previously, aside from selecting loci with topologically congruent gene trees, the same methods were used as when the entire data set was analyzed.

#### Estimating $t$ with topologically congruent branches

A novel approach was implemented where the estimate of $t$ for each branch in the species tree was the mean estimate for the equivalent branch across estimated gene trees. By only estimating $t$ in the species tree from gene tree branches that were equivalent to a branch in the species tree, this method only incorporated parts of gene trees that were topologically congruent with the species tree.

For this method, $t$ for individual gene trees was first estimated in RevBayes. Parameter estimation was performed in the same manner as for the species trees outlined above. The root age of the gene trees was fixed to the root age of the simulated species tree.

**Figure 4.**
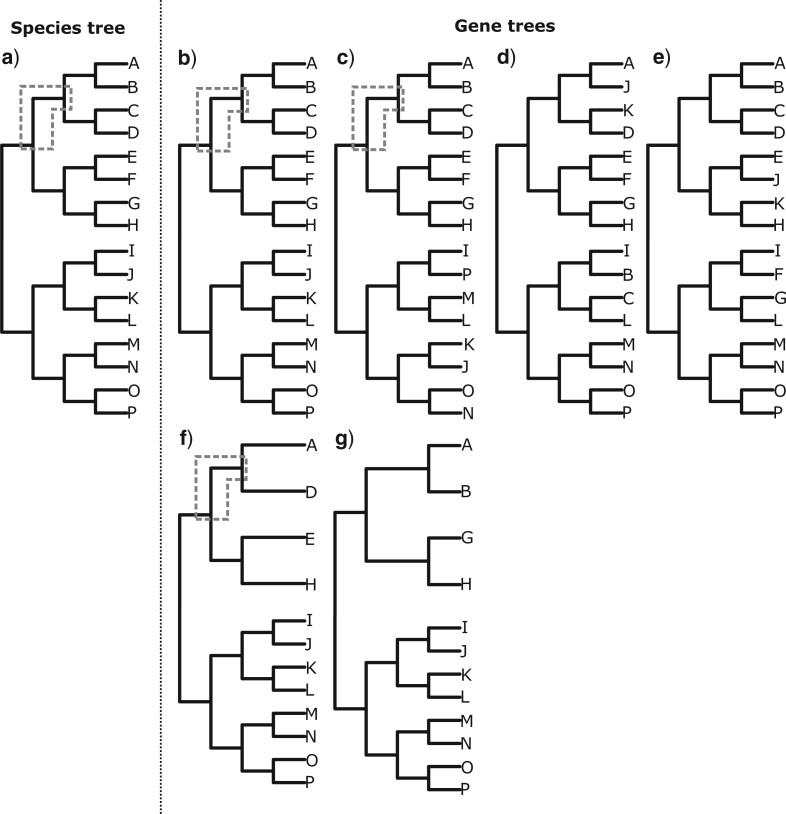
An illustration of how equivalent branches are identified in gene trees in order to estimate parameters for branches in the species tree. a) shows a species tree, and the gray dash-line box encloses the branch in the species tree for which equivalent branches are going to be identified in the gene trees. In b), the gene tree is identical to the species tree. There is therefore an equivalent branch, enclosed in the gray dash-line box. In c), there are topological differences between the gene tree and species tree. However, there is still an equivalent branch in the gene tree, enclosed by the gray dash-line box, where the descendant and ancestral nodes define the same clades as the descendant and ancestral nodes of the branch in the species tree. The gene trees shown in d) and e) do not have equivalent branches, because there is no branch in these gene trees where the descendant and ancestral nodes define the same clades as the descendant and ancestral nodes of the branch in the species tree. In f) and g), there is incomplete taxon sampling in the gene trees. In f), there is an equivalent branch in the gene tree (enclosed by the gray dash-line box) where the descendant and ancestral nodes define the same clades as the descendant and ancestral nodes of the branch in the species tree. In g), there is not an equivalent branch in the gene tree.

Branches in the gene trees that were equivalent to branches in the species tree were then identified. For a given branch in the species tree (Fig. 4a), an equivalent branch in a gene tree was defined where the clades descended from the descendent node and ancestral node of the branch were the same in the gene tree and species tree (Fig. 4b and c). By contrast, if the clades were not the same, the branch was not classed as equivalent (Fig. 4d and e). Incomplete taxon sampling in gene trees was accounted for such that if the sampled taxa in the gene tree defined the same clade as in the species tree, the branch could be classed as equivalent (Fig. 4f), but if they did not define the same clade the branch was not classed as equivalent (Fig. 4g).

This definition of equivalent branches is stricter than the definition used to identify incongruent branches in the rest of the article. This is necessary because it would be invalid to use the branch leading to the clade of ABCD in Figure 4e as a basis for making parameter estimates for the branch leading to the clade of ABCD in the species tree (Fig. 4a)—the branch in the gene tree is initiated by the divergence between a different pair of clades compared to the species tree and is not therefore the same branch.

Overall, the method cycled through each branch in the species tree, and for each branch determined which gene trees possessed an equivalent branch. The final estimate of $t$ for the branch in the species tree was then the mean estimate across all gene trees that possessed an equivalent branch. By contrast, if a gene tree did not possess an equivalent branch, estimates of $t$ for the branch in the species tree were not influenced by that gene tree.

When using this method, it is necessary to recognize that branches in gene trees are conceptually distinct from those in the species tree, even if the gene tree is topologically congruent with the species tree. This issue is perhaps most obvious in the context of a multispecies coalescent process, where branching events in gene trees always predate those in the species tree. These differences have several implications.

First, it is questionable whether it is even valid to derive branch-specific parameter estimates in the species tree directly from a mean estimate across all gene trees. Second, the implementation of temporal calibrations is difficult. Divergence times in the gene trees are known to differ from those in the species tree, yet when estimating gene trees, a root node calibration was used that was equal to the root age of the simulated species tree. Thus, the individual gene trees were technically calibrated to the incorrect age. This would also be an issue for the implementation of fossil calibrations in empirical data sets because fossil calibrations are implemented as evidence of timing for species divergences, despite the fact it is gene trees that are directly handled with this method. A further methodological difficulty concerns combining estimates of $t$ from time-calibrated gene trees into a single time-calibrated species tree. Different gene trees possess different combinations of branches that are equivalent to branches in the species tree, and when combining estimates of $t$ for these branches into a single time-calibrated species tree, it can mean that the tip times in the time-calibrated species tree are not aligned to the present. A final related issue is the generation of confidence intervals or posterior distributions for divergence time estimates in the species tree. The parameter estimates (and their associated uncertainty) upon which this method is based refer strictly to gene trees, making it conceptually problematic to then use these estimates as a basis for quantifying uncertainty in divergence time estimates in the overall species tree.

Although the description here focuses on the estimation of $t$, this method can also be used for the estimation of $n$ or $r$. The conceptual issues outlined above would still be relevant in many cases, although estimates of $n$ and $r$ can in practice be combined into a single species tree more easily than estimates of $t$.

#### Estimation of t in a multispecies coalescent framework

For data from the simple four-taxon simulation, a Yule process was used as the species tree prior with the root age fixed to 1. Because the gene trees were not simulated according to a multispecies coalescent process, the “correct” *Ne* could not be specified. Therefore, when performing the analysis *Ne* was sampled from an exponential distribution with *rate* $= 1$. A JC model of sequence evolution alongside a strict clock with $r = 0.05$ (the correct value) was used for each locus.

For analysis of data simulated under a multispecies coalescent process, the data were from the four-taxon multispecies coalescent simulation to enable computational tractability. The species tree prior was a Yule process with the root node fixed to 0.4 (the correct value). *Ne* was fixed to 0.12 (the correct value). As above, a JC model of sequence evolution alongside a strict clock with $r = 0.05$ (the correct value) was used for each locus.

The data set simulated under a four-taxon multispecies coalescent process was also used to estimate $t$ where; all simulated loci were analyzed as a concatenated alignment; all loci with topologically congruent gene trees were analyzed as a concatenated alignment; and congruent branches from each gene tree were analyzed. This enabled clarification that no unexpected patterns occurred when estimating $t$ with data from a multispecies coalescent simulation with a smaller species tree. This in turn provided a basis to compare the effectiveness of analyzing data in a multispecies coalescent framework to analyzing loci with topologically congruent gene trees or analyzing topologically congruent branches.

### The Implications of Topological Incongruence in an Empirical Data Set

A phylogeny for seed plants was estimated that incorporated at least one sample for each order. The final phylogeny comprised 103 tips, with 101 species representing 69 orders plus two lycophyte outgroups (*Isoetes tegetiformans* and *Selaginella apoda*). This provided a basis for determining the implications of topological incongruence and the effectiveness of methods for alleviating its impact in an empirical setting (Table 1).

The analysis focused on the Angiosperms353 loci, a set of 353 protein-coding genes that are thought to be mostly single-copy across angiosperms (Johnson et al. 2019). Exon sequences of 42 samples were obtained from Johnson et al. (2019) and the remainder were extracted from the One Thousand Plants Transcriptome Initiative (1KP) public database (Matasci et al. 2014; Carpenter et al. 2019; One Thousand Plant Transcriptomes Initiative 2019; http://www.onekp.com/public_data.html). blastn (Camacho et al. 2008) was used to match 1KP SOAPdenovo assemblies against the Angiosperms353 target sequences with a similarity threshold of $e = 1e^{-5}$ (Johnson et al. 2019). The nucleotide sequence data obtained above were then combined based on each gene id, and the matrix was aligned using MAFFT v7.471 (Katoh and Standley 2013) and cleaned using trimAl v1.4 (see code: https://github.com/pebgroup/Seed_Plant_BackBone) and the “-cons 60” option (Carpenter et al. 2019). Two genes (g6514 and g6886) were excluded because of low taxon coverage ($<$20$\%$), thus 351 genes remained for downstream analyses.

RAxML-NG (Kozlov et al. 2019) was run for each of the 351 gene alignments with 25 randomized parsimony starting trees for maximum likelihood (ML) tree search and 1000 nonparametric bootstrap (BS) replicates. The resulting ML tree from each gene was then rerooted by Phyx (Brown et al. 2017; if the outgroup sample was present) or Minimal Ancestor Deviation (Tria et al. 2017; if the outgroup was absent), and nodes with BS support below 10$\%$ were collapsed (Mirarab 2019). Lastly, the species tree was summarized from the 351 rooted gene trees using ASTRAL-III (Zhang et al. 2018).

Following the removal of the lycophyte outgroups, this species tree topology was used as a basis for estimating several different time-calibrated phylogenies in treePL v.1.0 (Smith and O’Meara 2012). In the first set of analyses, no fossil calibrations were implemented in the phylogeny, aside from the age of the root node being fixed at 350 Ma.

In this first set of analyses, three different input trees were used in which branch lengths in units of $n$ were estimated in different ways. First, branch lengths were estimated in RAxML from the entire data set, with all loci being concatenated, a single GTR $+$ G $+$ I model of sequence evolution being used across the alignment, and the topology fixed to that of the estimated species tree. Second, branch lengths were also estimated in RAxML but only loci for which the gene tree did not have any well-supported topological incongruence with the species tree (defined as any incongruent clade with BS support of $>$75) were used. This corresponded to 23 loci, each of which incorporated on average 85 taxa and an alignment length of 831 base pairs (the selected loci and corresponding gene trees can be found in the Supplementary material available on Dryad at http://dx.doi.org/10.5061/dryad.zw3r2287m and Github). Third, branch lengths in the species tree were estimated from the mean branch length estimate across all gene trees that were topologically congruent for each branch in the species tree. In this analysis, the selection of topologically congruent branches is carried out to enable estimation of $n$ prior to analysis in treePL, rather than to enable estimation of $t$ (as occurred in the simulations). These estimates of $n$ can be combined into a single species tree topology (unlike estimates of $t$). Therefore, in this case, the selection of topologically congruent branches can be used during the estimation of a single time-calibrated phylogeny. When selecting loci with topologically congruent gene trees, or selecting topologically congruent branches, incomplete sampling in the gene trees was accounted for, provided that the sampled tips defined the same clades in the gene tree and species tree, as shown in Figure 4.

For these three input trees, a smoothing value of 10,000 was used. Using no fossil calibrations and the same smoothing value enabled a straightforward comparison of the effects of topological incongruence for divergence time estimates with each input tree. This set of analyses is referred to as analyses with *simple* assumptions.

In a subsequent set of analyses, the same input trees were used, but 21 fossil calibrations were implemented as minimum constraints throughout the phylogeny, in addition to the root age constraint (Appendix S1 of the Supplementary material available on Dryad). Cross-validation analyses were also performed to determine a smoothing value for each tree and thus make different assumptions about among-branch-variation in $r$ for each tree. For the full data set, a smoothing value of 1 was used, with only topologically congruent loci a smoothing value of 10 was used, and with only topologically congruent branches a smoothing value of 0.1 was used. This subsequent set of analyses enabled the implications of topological incongruence to be determined in an environment that more closely reflects how divergence time analyses are typically performed. This set of analyses is referred to as analyses with *complex* assumptions.

## Results

### A General Theoretical Overview of the Implications of Topological Incongruence

#### Simple four-taxon simulations

**Figure 5.**
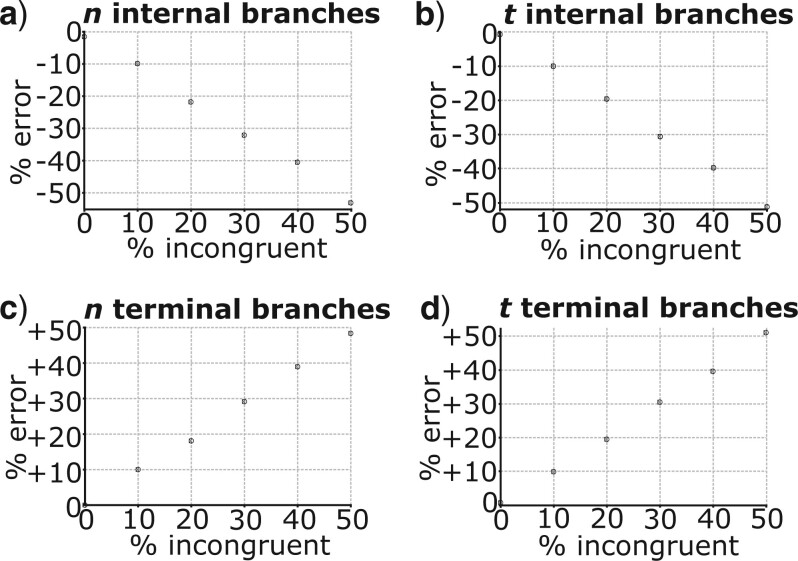
Percentage error for mean posterior estimates of $n$ and $t$ in analysis of data from the simple four-taxon simulations where all simulated data were analyzed, and different percentages of the gene trees were topologically incongruent with the species tree. The correct species tree topology was the same as that shown for the species tree in Figure 1, and the topologically incongruent gene trees had the same topology as shown in Figure 1. a) Refers to estimates of $n$ for internal branches in the species tree. b) Refers to estimates of $t$ for internal branches in the species tree. c) Refers to estimates of $n$ for terminal branches in the species tree. d) Refers to estimates of $t$ for terminal branches in the species tree.

With a higher percentage of gene trees that were topologically incongruent with the species tree, parameters for internal branches (all branches that are not terminals, and therefore those branches that were affected by topological incongruence) in the species tree were underestimated to a greater extent (Fig. 5a and b, Fig. S1a and b of the Supplementary material available on Dryad). The degree of underestimation was roughly equal to the percentage of topologically incongruent gene trees, such that with no topologically incongruent gene trees, $n$, $t$, and $r$ (using a relaxed clock with high variance) were not underestimated, whilst when 50$\%$ of gene trees were topologically incongruent, all three parameters were underestimated by approximately 50$\%$ (Fig. 5a and b, Fig. S1a of the Supplementary material available on Dryad). When $r$ was estimated using a relaxed clock with low variance, estimates only fell marginally below the correct value (Fig. S1b of the Supplementary material available on Dryad).

By contrast, for terminal branches, a higher percentage of topologically incongruent gene trees caused an inverse of the pattern for internal branches (Fig. 5c and d, Fig. S1c and d of the Supplementary material available on Dryad). Nonetheless, $t$ for terminal branches was always overestimated slightly more than $n$ or $r$ (Fig. 5c and d, Fig. S1c and d of the Supplementary material available on Dryad).

#### Simple 16-taxon simulations

**Figure 6.**
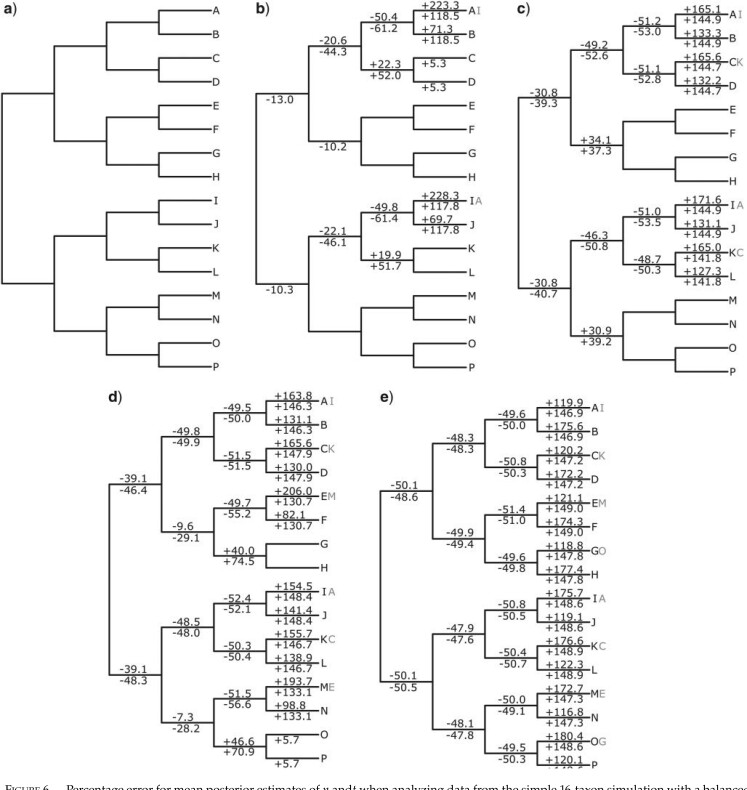
Percentage error for mean posterior estimates of $n$ and$t$ when analyzing data from the simple 16-taxon simulation with a balanced species tree and where all simulated data is analyzed. Numbers above branches refer to estimates of $n$, and numbers below branches refer to estimates of $t$. When the percentage error is below 5$\%$, no value is shown. The tree displayed refers to the species tree. Two tip labels indicate where the topology of the topologically incongruent gene tree differs from the topology of the species tree, with the gray right-hand label referring to the topologically incongruent gene tree. In all cases 50$\%$ of gene trees have evolved according to the incongruent gene tree topology, and 50$\%$ of gene trees have evolved according to the species tree topology. a) represents a special case where the incongruent gene tree is identical to the species tree. The incongruent gene trees for b–e) exhibit sequentially higher levels of topological incongruence.

As before, parameters for branches in the species tree that were affected by topological incongruence with gene trees were underestimated. However, the proportion of terminals that differed between the gene tree and species tree for a given clade in the species tree affected this pattern. For example, in Figure 6b and Figure S2b of the Supplementary material available on Dryad, there is topological incongruence for the branches in the species tree that lead to the clade comprising AB and the clade comprising IJ, and $n$, $t$, and $r$ (when estimated using a relaxed clock with high variance) for these branches was underestimated by at least 50$\%$. In Figure 6b and Figure S2b of the Supplementary material available on Dryad, there is also topological incongruence for the branches in the species tree that lead to the clades comprising ABCD, IJKL, ABCDEFGH, and IJKLMNOP. However, the proportion of terminals in these clades that differ between the species tree and gene trees is lower compared to the clades comprising AB and IJ, and the extent to which parameter values were underestimated for the deeper branches that subtend these clades was lower. When the proportion of terminals that differed for these larger clades increased (Fig. 6c–e, Fig. S2c–e of the Supplementary material available on Dryad), parameter values for the deeper branches subtending these clades were underestimated to a greater extent.

**Figure 7.**
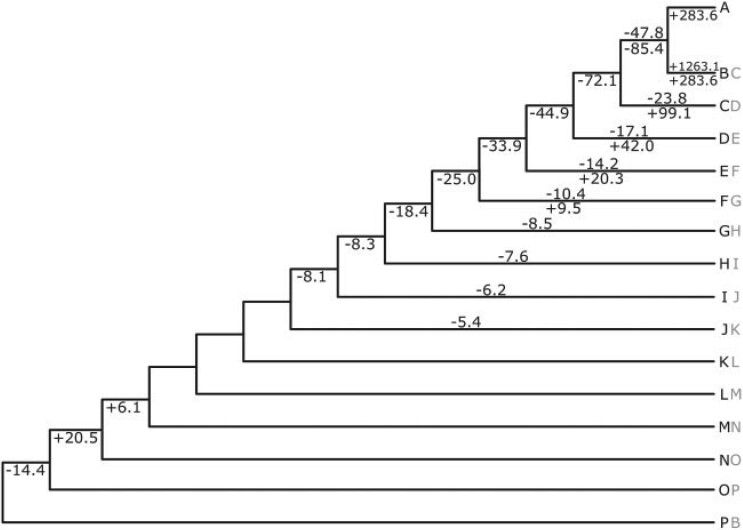
Percentage error for mean posterior estimates of $n$ and $t$ when analyzing data from the simple 16-taxon simulation with an imbalanced species tree and where all simulated data is analyzed. Numbers above branches refer to estimates of $n$, and numbers below branches refer to estimates of $t$. When the percentage error is below 5$\%$, no value is shown. The tree displayed refers to the species tree. Two tip labels indicate where the topology of the topologically incongruent gene tree differs from the topology of the species tree, with the gray right hand tip label referring to the topologically incongruent gene tree. In all cases, 50$\%$ of gene trees have evolved according to the incongruent gene tree topology, and 50$\%$ of gene trees have evolved according to the species tree topology.

The importance of the proportion of terminals that differ for a given clade was also highlighted by the imbalanced phylogeny, where there was a reduction in parameter underestimation for deeper branches in the species tree (Fig. 7, Fig. S3 of the Supplementary material available on Dryad). These deeper branches subtended clades with a lower proportion of tips that differed in the incongruent gene trees.

In both the balanced tree and imbalanced tree, there were important differences between estimates of $n$ and $r$ (when estimated with a relaxed clock with high variance), compared to estimates of $t$. For example, in the balanced tree, when only a low proportion of terminals differed between clades in the species tree and clades in the incongruent gene tree, $t$ for deeper branches was underestimated considerably more than $n$ or $r$ (Fig. 6b–d, Fig. S2b–d of the Supplementary material available on Dryad). By contrast, when a higher proportion of terminals differed, $t$, $n$, and $r$ were similarly underestimated (Fig. 6e, Fig. S2e of the Supplementary material available on Dryad). Likewise, in the imbalanced tree, deeper branches subtended clades with a lower proportion of tips that differed in the incongruent gene trees. At these deeper branches, $t$ was underestimated considerably more than $n$ or $r$ (Fig. 7, Fig. S3 of the Supplementary material available on Dryad). However, in all analyses when $r$ was estimated with a relaxed clock with *low* variance, estimates for $r$ only ever differed marginally from the correct value (Figs. S2 and S3 of the Supplementary material available on Dryad).

Parameter underestimation for branches affected by topological incongruence was also associated with “compensatory” parameter overestimation on branches that were not directly affected by topological incongruence. As with the four-taxon example, this occurred on terminal branches (Figs. 6 and 7, Figs. S2 and S3 of the Supplementary material available on Dryad), yet it also occurred on sister branches of those directly affected by topological incongruence. For example, in Figure 6b and Figure S2b of the Supplementary material available on Dryad, parameter values for the branch subtending the clade comprising CD in the species tree are overestimated.

As was the case with parameter underestimation, there were notable differences in parameter overestimation for $n$ and $r$ (when estimated with a relaxed clock with high variance) compared to $t$. First, for sister pairs of terminal branches, the overestimation of $t$ typically lay at the midpoint between the overestimation of $n$ and $r$, which tended to differ markedly between sister branches (Fig. 6b–e, Fig. S2b–e of the Supplementary material available on Dryad). Further, in the imbalanced species tree, $n$ and $r$ were often underestimated for terminal branches, whilst $t$ was overestimated (Fig. 7, Fig. S3 of the Supplementary material available on Dryad).

#### Multispecies coalescent simulation

For simplicity, only the percentage of gene trees that are topologically incongruent for a branch in the species tree is accounted for. The proportion of tips whose placement differs between the species tree and gene tree is not considered.

**Figure 8.**
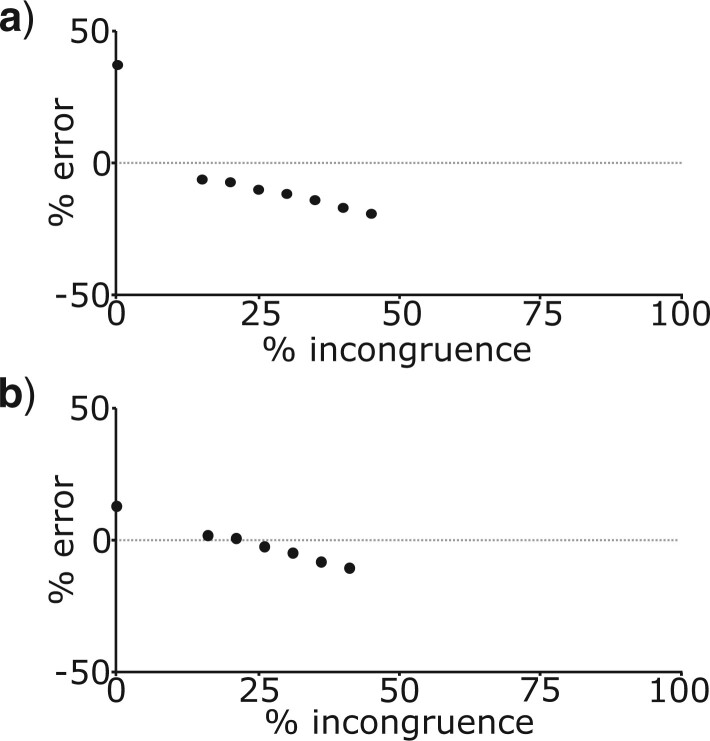
Percentage error of mean posterior estimates of $t$ for branches in the species tree, plotted against the percentage of gene trees that are topologically incongruent for that branch, when analyzing data from the multispecies coalescent 16 taxon simulations. These plots summarize results across 50 replicated experiments when all simulated data is analyzed. The black points represent the mean percentage error in estimates of $t$ for a given level of topological incongruence, with topological incongruence in bins of 5$\%$. a) refers to the balanced species tree and b) refers to the imbalanced species tree.

When the simulated species tree was either balanced (Fig. 2a) or imbalanced (Fig. 2f), a higher percentage of topologically incongruent gene trees for a given branch in the species tree resulted in greater underestimation of $t$ (Fig. 8). By contrast, $t$ for terminal branches (indicated by the plotted points with no incongruence in Fig. 8), was significantly overestimated in both the balanced and imbalanced species tree.

### Evaluating Methods for Alleviating the Effects of Topological Incongruence on Divergence Time Estimates

#### Estimating t with loci derived from gene trees that are topologically congruent with the species tree

This led to reduced error in estimates of $t$ in the species tree. This was the case when analyzing data from the simple four-taxon simulations, where estimates of $t$ were only minimally underestimated for internal branches, and only minimally overestimated for terminal branches (Table 2). Likewise, with this method estimates better approximated the correct value when analyzing data from the multispecies coalescent 16-taxon simulation, although considerable error did remain (Table 2).

**Table 2.** Comparison of estimates of $t$ in species trees when the entire data set is analyzed as a concatenated alignment, to when loci with gene trees that have different degrees of topological incongruence with the species tree are incorporated into the analysis

				95$\%$		$\%$ with correct	
		$\%$ error		HPD width		value in HPD	
Data	Inference method	Internal	Terminal	Internal	Terminal	Internal	Terminal
Simple four-taxon simulation	All loci concatenated	–51.2	+51.2	—	—	—	—
	Loci with entirely topologically congruent gene trees concatenated	–1.3	+1.3	—	—	—	—
Multispecies coalescent 16-taxon simulation	All loci concatenated	–11.1	+37.1	5.8	3.1	2.0	0.0
	Loci with entirely topologically congruent gene trees concatenated	–10.2	+21.8	15.2	10.2	27.0	0.0
	Loci with maximum of one topologically incongruent branch in gene tree concatenated	–10.2	+26.8	9.1	5.9	10.7	0.0
	Loci with maximum of two topologically incongruent branches in gene tree concatenated	–10.3	+29.9	7.1	4.5	3.7	0.0
	Loci with maximum of three topologically incongruent branches in gene tree concatenated	–10.5	+32.8	6.3	3.9	1.7	0.0

When analyzing data from the multispecies coalescent 16-taxon simulation, the effect of selecting loci with topologically congruent gene trees on node age estimates was also determined, given that node age estimates are typically of more direct interest in studies that involve divergence time estimation. This showed that for nodes subtending internal branches, there was a greater reduction in error compared to estimates of $t$ for internal branches (compare Tables 2 and 3).

**Table 3.** Comparison of node age estimates in species trees when the entire data set from the 16-taxon multispecies coalescent simulation is analyzed as a concatenated alignment, to when loci with gene trees that have different degrees of topological incongruence with the species tree are incorporated into the analysis

			95$\%$		$\%$ with correct	
	$\%$ error		HPD width		value in HPD	
Inference Method	Subtending internal	Subtending terminal	Subtending internal	Subtending terminal	Subtending internal	Subtending terminal
All loci concatenated	+9.4	+37.1	1.8	3.5	14.2	0.0
Loci with entirely topologically congruent gene trees concatenated	+2.4	+21.8	5.3	10.2	52.3	0.0
Loci with maximum of one topologically incongruent branch in gene tree concatenated	+4.6	+26.3	3.4	5.9	27.7	0.0
Loci with maximum of two topologically incongruent branches in gene tree concatenated	+6.3	+29.9	2.3	4.5	16.0	0.0
Loci with maximum of three topologically incongruent branches in gene tree concatenated	+7.7	+32.8	2.0	3.9	14.6	0.0

Analyses based on the 16-taxon multispecies coalescent simulation demonstrated how analyzing loci from topologically congruent gene trees led to a trade-off between ameliorating the effect of topological incongruence and the precision of parameter estimates. This is because only analyzing loci with topologically congruent gene trees resulted in considerably less precise estimates (Tables 2 and 3). If the threshold for excluding incongruent gene trees was sequentially weakened, such that gene trees with sequentially higher levels of topological incongruence were incorporated into the analysis, estimates became more precise. However, the percentage error also increased and the 95$\%$ highest posterior density (HPD) was less likely to include the correct value (Tables 2 and 3). Nevertheless, for estimates of $t$ for branches, the 95$\%$ HPD was unlikely to include the correct value even when only loci from topologically congruent gene trees were analyzed (Table 2).

#### Estimating t with topologically congruent branches

This also led to reduced error in estimates of $t$ in the species tree. When analyzing data from the simple four-taxon simulation and multispecies coalescent 16-taxon simulation, it had a similar impact on estimates compared to when only loci with topologically congruent gene trees were analyzed, although the effects were even more pronounced (Table 4). Due to difficulties in combining estimates from different gene trees, node age estimates were not calculated with this method.

**Table 4.** Comparison of estimates of $t$ for branches in the species tree when the entire data set is analyzed as a concatenated alignment, to when only topologically congruent branches from gene trees are used for parameter estimation

		$\%$ error	
Data	Inference method	Internal	Terminal
Simple four-taxon simulation	All loci concatenated	–51.2	+51.2
	Topologically congruent branches	–2.5	+2.3
Multispecies coalescent 16-taxon simulation	All loci concatenated	–11.1	+37.1
	Topologically congruent branches	+3.2	+5.9

#### Estimating t in a multispecies coalescent framework

When analyzing data from the simple four-taxon simulation, considerable error remained in estimates of $t$ (Table 5). With the analysis of data from the multispecies coalescent simulation, error in estimation of $t$ was almost eliminated (Table 5), with the method being considerably more effective than selecting loci with topologically congruent gene trees or selecting topologically congruent branches (Table 5). For analyses in this section, error in node age estimates is not summarized because it is equal to error in estimates of $t$ for terminal branches.

**Table 5.** Comparison of estimates of $t$ when the entire data set is analyzed as a concatenated alignment, to when the data set is analyzed in a multispecies coalescent framework

		$\%$ error	
Data	Inference method	Internal	Terminal
Simple four-taxon simulation	All loci concatenated	–51.2	+51.2
	All loci analyzed as multispecies coalescent	–10.0	+10.0
Multispecies coalescent four-taxon simulation	All loci concatenated	–50.6	+50.6
	All loci analyzed as multispecies coalescent	–1.25	+1.25
	Loci with entirely topologically congruent gene trees concatenated	–8.3	+8.3
	Topologically congruent branches	–15.4	+9.4

*Note*: To enable comparison, where data has been simulated under a multispecies coalescent framework, estimates of $t$ are also shown where only topologically congruent gene trees or topologically congruent branches are analyzed

### The Implications of Topological Incongruence in an Empirical Data Set

**Figure 9.**
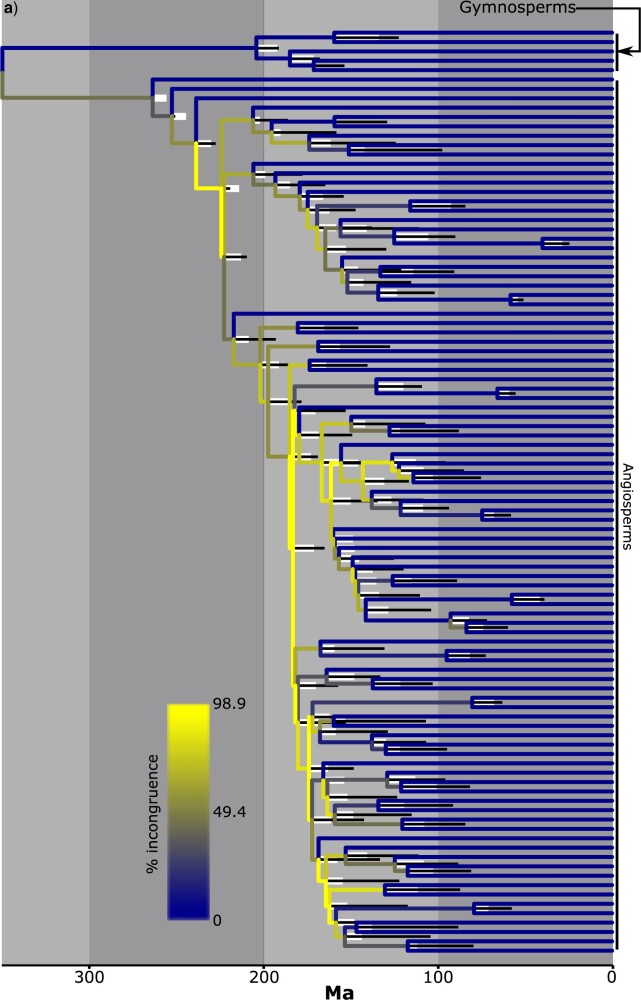

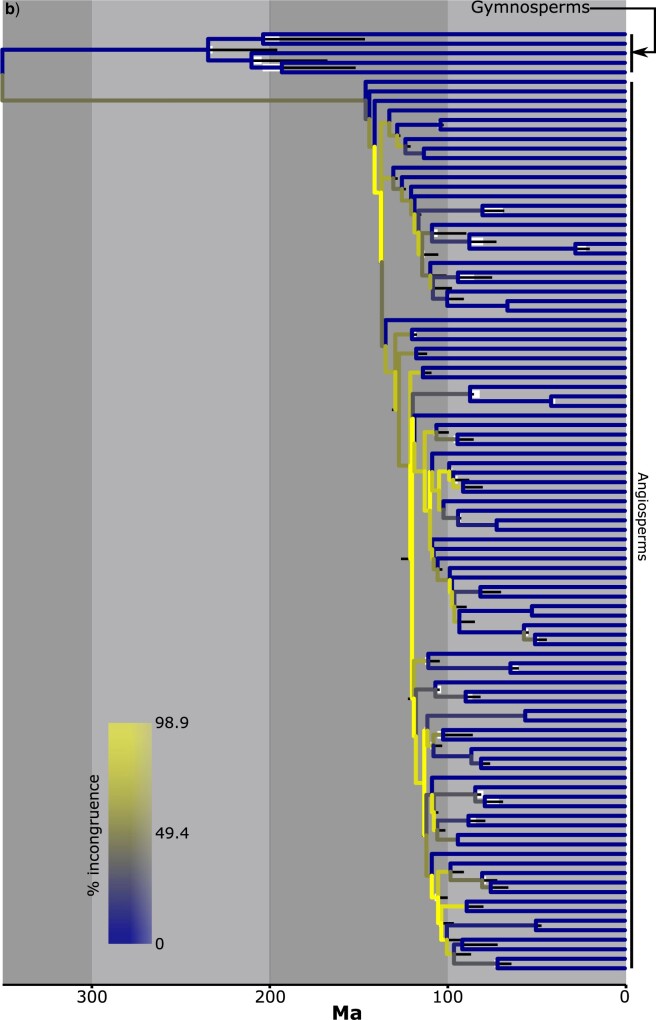
Time-calibrated phylogenies for seed plants based on a data set of 351 single-copy loci. Branch colors represent the percentage of gene trees that are topologically incongruent for a given branch. In a), simple assumptions are used: the root node is fixed to 350 Ma, no other calibrations are implemented, and minimal among-branch-variation in $r$ is assumed. In b), complex assumptions are used: 21 fossil calibrations are implemented (Appendix S1 of the Supplementary material available on Dryad) as minimum constraints, and cross-validation analysis is performed in treePL to determine the degree of among-branch-variation in $r$ that should be assumed when inferring the time-calibrated phylogeny. In each case, the visible time-calibrated phylogeny is inferred with all 351 loci. For each node, the thick white line extends to the age estimate for the clade when only loci with topologically congruent gene trees are analyzed, and the thin black line extends to the age estimate for the clade when only topologically congruent branches are analyzed.

The key patterns revealed in the simulations were replicated in the empirical analyses. For example, only analyzing loci from topologically congruent gene trees, or only analyzing topologically congruent branches, resulted in lower estimates of $t$ for terminal branches in the species tree with no topological incongruence, and higher estimates of $t$ for internal branches that were affected by topological incongruence (Fig. 9; Table 6).

**Table 6.** A comparison of estimates of $t$ for branches in the empirical example when different methods are used to account for topological incongruence

		$\%$ difference to all data analyzed as a concatenated alignment	
Assumptions$^{\rm a}$	Inference method	Internal	Terminal
Simple	Loci with topologically congruent gene trees	–1.2	–9.4
	Topologically congruent branches	+274.1	–24.0
Complex	Loci with topologically congruent gene trees	+1.1	–1.9
	Topologically congruent branches	+634.6	–7.8

$^{\rm a}$ *Simple* assumptions used a smoothing value of 10,000 and no internal fossil calibrations, *complex* assumptions used a smoothing value selected by cross-validation and internal fossil calibrations.

Notably, this effect was more pronounced when only analyzing topologically congruent branches, especially for internal branches (Fig. 9; Table 6). This difference may stem from the incorporation of some loci with topologically incongruent gene trees in the analysis that is intended to be based on loci with topologically congruent gene trees, in cases where gene trees contain topological incongruence with a BS support of $<= 75$ (see Materials and Methods section).

Further, the pattern for internal branches was somewhat distorted because many of these branches are very short (see Supplementary material available on Dryad and Github). As such, stochasticity in the substitution process, alongside small changes in absolute parameter estimates leading to very large percentage differences, led to some unexpected patterns. This explains the 1.2$\%$ mean reduction in estimates of $t$ for internal branches estimated with loci from topologically congruent gene trees (when using simple assumptions), and potentially the apparently drastic difference between the two methods at internal branches (Table 6).

The effects on estimates of $t$ in the empirical example had a marked impact on node age estimates in the species tree, with the lengthening of internal branches and shortening of terminal branches tending to cause younger node age estimates throughout the species tree (Fig. 9; Table 7). Nonetheless, the percentage difference for node age estimates was considerably smaller compared to estimates of $t$ (compare Tables 6 and 7). This is because estimates of $t$ often correspond to very short branches where small absolute changes lead to very large percentage differences. Further, the effects for node age estimates, and to some extent estimates of $t$, were more clearly apparent in the analyses with simple assumptions (Fig. 9; Tables 6 and 7).

**Table 7.** A comparison of node age estimates in the empirical example when different methods are used to account for topological incongruence

		$\%$ difference to all data analyzed as a concatenated alignment	
Assumptions$^{\rm a}$	Inference method	Subtending internal	Subtending terminal
Simple	Loci with topologically congruent gene trees	–6.8	–10.5
	Topologically congruent branches	–18.4	–26.9
Complex	Loci with topologically congruent gene trees	–0.6	–2.4
	Topologically congruent branches	–3.3	–9.9

$^{\rm a}$ *Simple* assumptions used a smoothing value of 10,000 and no internal fossil calibrations, *complex* assumptions used a smoothing value selected by cross-validation and internal fossil calibrations.

## Discussion

### Topological Incongruence Leads to Error in Estimates of Times and Substitution Rates in the Species Tree

#### Error in estimation of the number of substitutions underpins error in estimation of times and substitution rates

Topological incongruence between gene trees and the species tree led to underestimation of the number of substitutions ($n$), times ($t$), and substitution rates ($r$) for branches in the species tree that were directly affected by topological incongruence. This result was consistent across all analyses of simulated data (Figs. 5a and b, 6, 7, and 8, Figs. S1a and b, S2, and S3 of the Supplementary material available on Dryad). For data from the simple four-taxon simulation, topological incongruence had an approximately linear effect on parameter underestimation for internal branches (those directly affected by topological incongruence). As such, the percentage of gene trees that were topologically incongruent corresponded to the degree to which $t$, $n$, and $r$ were underestimated (Fig. 5a and b, Fig. S1a of the Supplementary material available on Dryad). For data from the simulations based on larger species trees, the patterns were more complex because for a given branch in the species tree, topological incongruence relates to the percentage of gene trees that are topologically incongruent *and* the proportion of terminals that differ between the gene tree and species tree for a given clade (Figs. 6 and 7, Figs. S2 and S3 of the Supplementary material available on Dryad). For example, in Figure 6, $t$ for the branch subtending the clade comprising ABCDEFGH in the species tree was only underestimated by 13$\%$ when one terminal differed in the relevant clade in the gene tree (Fig. 6b) but was underestimated by 48.6$\%$ when 4 terminals differed (Fig. 6e).

Analyses of simulated data also showed consistently that topological incongruence led to overestimation of $t, r$, and $n$ for branches in the species tree that were not directly affected by topological incongruence (Fig. 5c and d, 6, 7, and 8, Figs. S1c and d, S2, and S3 of the Supplementary material available on Dryad). With data from the simple four-taxon simulation, this resulted in overestimation of $t, r$, and $n$ for terminal branches (Fig. 5c and d, Fig. S1c and d of the Supplementary material available on Dryad), which by definition are not affected by topological incongruence. In the larger trees, all three parameters were not only overestimated for terminal branches but also for the sister branches of those directly affected by topological incongruence (Figs. 6–8, Figs. S2 and S3 of the Supplementary material available on Dryad).

These results can be explained according to the findings of Mendes and Hahn (2016). Specifically, underestimation of $t$, $r$, and $n$ for branches in the species tree that are directly affected by topological incongruence corresponds to Mendes and Hahn’s (2016) finding that substitutions cannot be assigned to branches in the species tree that do not exist in the underlying gene tree. Meanwhile, overestimation of $t$, $r$, and $n$ for branches not directly affected by topological incongruence corresponds to the finding from Mendes and Hahn (2016) that substitutions occurring on topologically incongruent gene trees are estimated to occur several times on the species tree. Aside from showing that the findings of Mendes and Hahn (2016), which relate specifically to $n$, lead to equivalent effects for $t$ and $r$, the analyses presented here also show that Mendes and Hahn’s findings are relevant when analyzing a concatenated alignment of multiple loci derived from differing percentages of topologically incongruent gene trees.

#### Error in estimation of $t$ and $r$ has distinct properties from error in estimation of $n$

Despite consistency with the findings of Mendes and Hahn (2016), estimation of $t$ and $r$ is distinct from the estimation of $n$, because $t$ and $r$ are nonidentifiable parameters meaning that estimates are necessarily constrained by assumptions. This was evident in analyses of data from the simple four-taxon simulations, where $t$ for terminal branches was overestimated to a greater extent than $n$ for terminal branches (Fig. 5c and d). This likely reflects the fact that the simulated dataset caused $n$ (and by extension $t$) for internal branches to be underestimated by $>$ 50$\%$. Therefore, when estimating $t$, where the tips must be sampled at the present and the root age is fixed, terminal branches were necessarily overestimated by $>$ 50$\%$. By contrast, estimation of $n$ is not constrained by such assumptions, meaning that estimation of $n$ for terminal branches did not need to compensate for the fact that $n$ for internal branches was underestimated by $>$ 50$\%$.

Important differences between $t$ and $n$ were also evident when analyzing data from the simple 16-taxon simulations, although the patterns were more complex. First, note the differences in estimates of $n$ compared to estimates of $t$ for sister pairs of terminal branches (Fig. 6). Where the placement of only a low proportion of terminals differed between the species tree and gene tree (Fig. 6b–d), $n$ for the terminal branch of the taxon that belonged to an entirely different clade in the incongruent gene tree was far higher. For example, in Figure 6b the terminal leading to A in the species tree is overestimated considerably more than the terminal leading to B. This is because synapomorphies for the incongruent gene tree clades AJKL and AJ are assigned to the terminal leading to A in the species tree. By contrast, although synapomorphies for the incongruent gene tree clade IB are assigned to the terminal leading to B in the species tree, many of the synapomorphies for the incongruent gene tree clade IBCD do not appear to be assigned to the terminals leading to BCD in the species tree. Instead, they are assigned to the branch leading to ABCD in the species tree, as evidenced by the fact that $n$ for this branch is only underestimated by 20.6$\%$, and $n$ for the terminals leading to C and D are only minimally overestimated. As topological incongruence increased, these patterns became more complex, and interdependencies among branches emerged (Fig. 6). Regardless, error in estimates of $t$ for sister pairs of terminal branches was necessarily intermediate between error in estimates of $n$, given that $t$ for the two terminal branches had to be identical (Fig. 6).

Alternatively, for internal branches in the balanced simple 16-taxon tree, $t$ was underestimated considerably more than $n$, especially with low or intermediate levels of topological incongruence (Fig. 6b–d). This is because with low or intermediate levels of topological incongruence, overestimation of $t$ for terminal branches, such as those leading to A and B, combined with the root age constraint, led to underestimation of $t$ for internal branches, even when $n$ for these internal branches was at most minimally underestimated. By contrast, with high incongruence (Fig. 6e), $n$ for the internal branches was significantly underestimated. In this case, underestimation of $t$ was underpinned by underestimation of $n$ (as opposed to the assumptions of the divergence time analysis), such that $n$ and $t$ were similarly underestimated.

Similarly complex interactions were observed in the imbalanced simple 16-taxon tree (Fig. 7). Note how $t$ for deeper internal branches (that subtend clades with a lower proportion of tips that differ in the incongruent gene trees) was underestimated to a greater extent than $n$. This likely stems from the massive overestimation of $t$ for the terminals leading to A and B combined with the fixed age of the root node. Alternatively, note how $n$ for some terminal branches was underestimated in the imbalanced 16-taxon tree (Fig. 7). $n$ for these branches was underestimated because the terminal branches have different values for $n$ in the simulated species tree compared to the simulated topologically incongruent gene trees, and some terminal branches in the simulated topologically incongruent gene trees have a lower $n$ than the terminal branch leading to the same taxon in the simulated species tree. However, $t$ for these same branches was still significantly overestimated given that the tips must be sampled at the present but diverge at a time prior to the divergence of the terminals leading to A and B.

The analyses of simulated data also demonstrated how estimation of $r$ is distinct from estimation of $n$, and that these differences are underpinned by the assumptions that are implemented when estimating $r$. With data from the simple four-taxon and simple 16-taxon simulations, and where $r$ was estimated with a relaxed clock with high variance, error in estimation of $r$ corresponded very closely to error in estimation of $n$ (Figs. 5–7, Figs. S1a and c, S2, and S3 of the Supplementary material available on Dryad). In this case, the relaxed clock exerted very little influence on estimates of $r$, and given that all divergence times were constrained to the correct value, variation in estimates of $r$ corresponded almost exactly to variation in estimates of $n$. By contrast, when $r$ was estimated with a relaxed clock that had a low variance, estimates of $r$ were constrained such that error in estimates of $r$ differed markedly from error in estimates of $n$ (Figs. 5–7, Figs. S1b, d, S2, and S3 of the Supplementary material available on Dryad). In this case, parameter estimates were strongly influenced by the assumptions of the relaxed clock model. Note that the result from this analysis, whereby estimates of $r$ only differed marginally from the correct value, is not justification for using relaxed clocks with low variances in empirical systems. This analysis is an artificial scenario where the correct divergence times and correct mean for the lognormal distribution of the relaxed clock are known. The purpose of the analysis is simply to illustrate the power of the assumptions that are exerted by the relaxed clock model.

Overall, these findings highlight how the assumptions that are incorporated into divergence time analyses modulate the effect that topological incongruence has on parameter estimates. As such, patterns for $t$ and $r$ do not always correspond to the patterns that are observed for $n$.

### The Undesirable Effects of Topological Incongruence Can Be Ameliorated

#### Selecting loci with congruent gene trees or selecting congruent branches

In a manner consistent with previous analyses (Jarvis et al. 2014; Doyle et al. 2015; Smith et al. 2018; Mongiardino Koch 2021), this study showed that analyzing subsets of loci with gene trees that are topologically congruent with the species tree (Fig. 3b) can help to ameliorate the effect of topological incongruence on divergence time estimation (Tables 2 and 3). Likewise, only taking into account topologically congruent branches in each gene tree (Fig. 3c) was similarly (if not more) effective (Table 4). These results were consistent across all analyses, although they were particularly pronounced when analyzing data from the simple four-taxon simulations, where error was almost eliminated (Tables 2 and 4).

#### Error remains when data is simulated in a multispecies coalescent framework

Despite the effectiveness of analyzing loci with topologically congruent gene trees, or analyzing topologically congruent branches, analyses of data simulated under a multispecies coalescent framework continued to be affected by considerable error in estimates of $t$ (Tables 2–4). This is because even though these methods necessarily remove the effect of topological incongruence, divergences in individual gene trees still predate divergences in the species tree (Angelis and dos Reis 2015). For terminal branches, whereby $t$ continued to be significantly overestimated (Tables 2 and 4), this effect is likely to be especially relevant. This is because even when loci from topologically congruent gene trees or topologically congruent branches are sampled, $t$ for terminal branches in the gene trees must always be greater than $t$ for terminal branches in the species tree. By contrast, for other branches, the branch in the gene tree may terminate earlier than the branch in the species tree, meaning that $t$ is not always larger in the gene tree than in the species tree.

The fixing of the root node to the correct age (as occurs in all the analyses presented here) alongside the consistent overestimation of $t$ for terminal branches, can explain the slight underestimation of $t$ for internal branches even when only loci with congruent gene trees or only congruent branches were sampled (Tables 2 and 4). Further, with the root node fixed to the correct age, node age estimates in the species tree were closer to the correct value compared to estimates of $t$ for branches. This likely stems from the fact that particularly for deeper nodes, the root node constraint prevented nodes from being significantly older than the correct value (Tables 2 and 3).

Regardless of this complexity, the overestimation of $t$ for terminal branches and underestimation of $t$ for internal branches were both greater when the full data set was analyzed (Tables 2 and 4). This highlights the importance of topological incongruence, and the value of selecting loci with topologically congruent gene trees (Fig. 3b) or topologically congruent branches (Fig. 3c).

It is nonetheless unsurprising that in an additional experiment where the data were both simulated and analyzed as part of a multispecies coalescent process, error in divergence time estimates was effectively eliminated (Table 5). In this case, divergence times were estimated in a framework that explicitly incorporates topological and temporal incongruence between the species tree and gene trees. Divergence time estimation in a multispecies coalescent framework can therefore be extremely valuable. However, there are two caveats. First, analyses in a multispecies coalescent framework are extremely computationally intensive, and large-scale analyses using this method are likely to require unrealistic resources. Second, analyses in a multispecies coalescent framework make their own important assumptions about the relationship between gene trees and the species tree that can negatively affect parameter estimates. For example, with data that were analyzed in a multispecies coalescent framework but not simulated in such a framework, there was considerable error in estimates of $t$ (Table 5). It may therefore be the case that selecting loci with topologically congruent gene trees (e.g., using SortaDate, Smith et al. 2018), or selecting topologically congruent branches, is a more feasible approach in many contexts.

#### Uncertainty and precision when only analyzing loci with congruent gene trees or only analyzing congruent branches

Although only analyzing loci with topologically congruent gene trees led to more accurate estimates of $t$ and node ages in the species tree, removal of data was associated with decreased precision in parameter estimates, as evidenced by the wider 95$\%$ HPD intervals (Tables 2 and 3). When loci with gene trees containing sequentially higher levels of topological incongruence were additionally incorporated into the analysis, parameter estimates were more precise (Tables 2 and 3), yet even incorporating loci with gene trees containing low levels of topological incongruence significantly reduced the probability that the correct value was included in the 95$\%$ HPD (Tables 2 and 3).

Nonetheless, even when only loci with topologically congruent gene trees were sampled, the 95$\%$ HPD was unlikely to contain the correct value for $t$ when analyzing data from the multispecies coalescent simulation (Table 2) (although it was considerably more likely to contain the correct value for node ages; Table 3). This is a result of temporal discordance between branching events in gene trees and species trees, thus reiterating the point that branching events in gene trees do not necessarily correspond to those in the species tree, and that methods that assume such concordance (as is common in divergence time estimation) should be used critically.

When only analyzing congruent branches, the conceptual distinction between gene tree branches and species tree branches made it difficult to present a single measure of uncertainty. The estimates of $t$ in the species tree are derived from separate analyses that are making inferences about different entities (branches in each gene tree). As such, estimates were not combined into a single posterior distribution with a 95$\%$ HPD. Estimates of $t$ from this method are therefore somewhat heuristic—although they are based on the analysis of entities (gene tree branches) that will often be affected by similar evolutionary processes (given they are from the same organism), these entities are distinct from each other and from branches in the species tree.

This conceptual issue, which stems from the implementation of necessarily simplistic assumptions about the relationship between gene trees and the species tree, is also relevant to other methods, such as parameter estimates in the species tree based on the analysis of concatenated alignments of individual loci. However, it is the fact that parameter estimates are explicitly derived from estimated gene trees that throws this issue into a particularly sharp light in this case. Nevertheless, estimating parameters from congruent branches in each gene tree still proves useful for determining the implications of topological incongruence between gene trees and the species tree—especially where there are no gene trees that are entirely congruent with the species tree.

### Characterizing the Implications of Topological Incongruence in an Empirical Setting

Only analyzing loci from topologically congruent gene trees, or only analyzing topologically congruent branches, demonstrated that topological incongruence has similar effects on divergence time estimates in an empirical setting (Fig. 9, Tables 6 and 7). In both cases, estimates of $t$ in the species tree for internal branches with topological incongruence were higher compared to when all loci were analyzed, whilst estimates of $t$ for terminal branches with no topological incongruence were lower compared to when all loci were analyzed (Fig. 9; Tables 6 and 7).

Further, it was apparent that in the empirical analyses with complex assumptions that reflect how divergence time analyses are typically performed, the effect of topological incongruence was weaker, because only analyzing loci with topologically congruent gene trees or analyzing topologically congruent branches had a smaller effect on parameter estimates (Fig. 9b; Tables 6 and 7). This finding is consistent with the analyses of simulated data which showed that methodological assumptions in divergence time estimation modulate the effect of topological incongruence on divergence time estimates.

The consistency of results between the analyses of empirical and simulated data is important for two key reasons. First, divergence time estimation in empirical settings is considerably more complex than the simulations, not least because of error and uncertainty in gene tree estimation which may distort perceptions of topological incongruence. Second, the correct values in the empirical analyses are unknown, meaning that comparison with consistent results from analyses of simulated data is crucial for making meaningful conclusions about the implications of incongruence in the empirical dataset.

### Approaching Divergence Time Estimation in the Presence of Topological Incongruence

#### Filtering for topologically congruent loci or branches

This study shows clearly that topological incongruence between gene trees and the species tree affects estimates of $n, t$, and $r$ in the species tree. As such we advocate methods that involve the selection of loci derived from gene trees that are topologically congruent with the species tree. However, for some data sets, there are no gene trees that are entirely congruent with the topology of the species tree (Salichos and Rokas 2013). In this instance, some loci would have to be incorporated into the analysis that have gene trees that are topologically incongruent with the species tree. This would be likely to have an impact on divergence time estimates. Where there are no gene trees that are entirely topologically congruent, we would therefore also advocate performing analyses that only incorporate topologically congruent branches from the gene trees. This can enable a useful comparison to explore the implications of topologically incongruent gene trees on divergence time estimates. However, we emphasize that the power of selecting topologically congruent branches is as a means for making comparative assessments, rather than as an approach that is likely to underpin divergence time estimates in the future. This is because it is difficult to incorporate uncertainty with this approach, and it can only be used for estimating a time-calibrated species tree with methods that require an input tree with branch lengths in units of $n$, such as treePL. Currently, such methods explore a relatively limited set of assumptions.

Nonetheless, we consider that such comparisons are important, because setting general limits on an acceptable level of topological incongruence to incorporate into an analysis is extremely challenging. We tentatively explored this in our analyses of simulated data, where we highlighted a trade-off between precision and accuracy when loci with gene trees incorporating different amounts of topological incongruence were incorporated into the analysis. However, the nature of this trade-off is likely to depend on several variables including; the extent of topological incongruence within the data set; the manner and accuracy by which topological incongruence has been estimated; and the methods used when estimating divergence times.

Topological incongruence may also be linked to other variables relevant to divergence time estimation. For example, in some data sets topologically incongruent genes trees may tend to be more “clock-like.” When selecting loci to incorporate into divergence time analyses, it is therefore also important to be aware of the nature of these potential interdependencies (Smith et al. 2018; Mongiardino Koch 2021).

#### The importance of conceptual distinctions between gene trees and the species tree

When assessing the implications of topological incongruence between gene trees and the species tree, it is also important to be aware of the conceptual distinctions between these two types of trees. This is because most approaches to divergence time estimation do not explicitly incorporate such distinctions, with analyses of concatenated alignments (even when loci with topologically incongruent gene trees are removed) or topologically congruent branches in gene trees assuming that evidence for gene tree divergence times corresponds directly to species tree divergence times. The implications of this simplistic approach were illustrated by the analysis of data from the multispecies coalescent simulation, where even when loci with topologically congruent gene trees or topologically congruent branches were used to estimate $t$, considerable error remained (Tables 2–4). Likewise, an inability to effectively incorporate conceptual distinctions between gene trees and species trees is pertinent to specific methodological components of divergence time estimation. This includes fossil calibrations, which despite being used as evidence of species tree divergence times, are used with molecular evidence that corresponds directly to gene trees.

Estimation of divergence times in a multispecies coalescent framework can incorporate a more complex relationship between gene trees and the species tree, and may therefore be valuable. However, this approach is computationally prohibitive in many data sets, and a multispecies coalescent framework is in itself an imperfect framework for associating gene trees with the species tree (e.g., Sukumaran and Knowles 2017).

Overall, it is therefore important that divergence time estimates are not interpreted in a manner that assumes precise conformity to the “true” species tree. Instead, they act as a framework for providing some evidence about when different clades may have evolved.

#### The effect of topological incongruence on divergence time estimates is indirect, and methodological assumptions are important

Despite the important effects of topological incongruence between gene trees and the species tree, its effect on estimates of $t$ is indirect. Instead, the direct effect of topological incongruence is on estimates of $n$. $n$ in turn provides a basis for estimation of $t$ (Britton 2005), but the effect that error in estimation of $n$ has on the estimation of $t$ is modulated by the additional assumptions required for divergence time estimation. In empirical data sets, this includes assumptions about the fossil record when implementing fossil calibrations (Near and Sanderson 2004; Ho and Phillips 2009; Magallón et al. 2013; 2015; Morris et al. 2018), and assumptions about variation in $r$ (Donoghue and Benton 2007; Magallón et al. 2013; Carruthers and Scotland 2020). Therefore, although accounting for topological incongruence is important, divergence time analyses are fundamentally sensitive to how these subsequent assumptions are implemented, and they will remain sensitive to these assumptions however well topological incongruence between gene trees and the species tree is accounted for.

## Data Availability

Code for simulations and divergence time estimation is available at: https://github.com/pebgroup/tree_incongruence_divergence_times. Code and data for phylogenetic inference in the empirical example is available at: https://github.com/pebgroup/Seed_Plant_BackBone.

## Supplementary Material

Data available from the Dryad Digital Repository: http://dx.doi.org/10.5061/dryad.zw3r2287m.
